# The Relationship Between Autistic Traits and Depression: The Chain Mediating Roles of Interpersonal Competence and Social Avoidance and Distress

**DOI:** 10.3390/bs15121658

**Published:** 2025-12-02

**Authors:** Yongsheng Wang, Guangyi Lv, Daoyi Liu, Xin Li, Peng Li, Xiaolei Gao

**Affiliations:** 1 Key Research Base of Humanities and Social Sciences of the Ministry of Education, Academy of Psychology and Behavior, Tianjin Normal University, Tianjin 300387, China; wangyongsheng@tjnu.edu.cn (Y.W.); lixinpsy@tjnu.edu.cn (X.L.); lipeng@tjnu.edu.cn (P.L.); 2 Faculty of Psychology, Tianjin Normal University, Tianjin 300387, China; lvguangyi2022@163.com (G.L.); liudaoyi1949@163.com (D.L.); 3 Tianjin Key Laboratory of Student Mental Health and Intelligence Assessment, Tianjin 300387, China; 4 Plateau Brain Science Research Center, Xizang University, Lhasa 850000, China

**Keywords:** autistic traits, depression, interpersonal competence, social avoidance and distress, chained mediation

## Abstract

Individuals with high autistic traits typically face a higher risk of depression, making it necessary to explore the relationship between autistic traits and depression in depth. Building on previous research, this study further investigates the roles of interpersonal competence, social avoidance and distress in the relationship between autistic traits and depression. A total of 674 college students were surveyed online using the Chinese version of the Autism-Spectrum Quotient (AQ) questionnaire, the Chinese version of the Interpersonal Competence Questionnaire (ICQ), the Chinese version of the Social Avoidance and Distress Scale (SAD), and the Chinese version of the 13-item Beck Depression Inventory (BDI). Correlation analysis results indicate that autistic traits exhibit a significant negative correlation with interpersonal skills, while showing a significant positive correlation with social distress, social avoidance, and depression levels. Conversely, interpersonal skills demonstrate a significant negative correlation with social avoidance, distress, and depression. Social avoidance and distress showed a significant positive correlation with depression. Chain mediation analysis revealed that interpersonal skills exerted a chain mediating effect between autistic traits and depression via social avoidance and distress. These findings provide insights for further exploration of the relationship and mechanisms underlying autistic traits and depression in individuals.

## 1. Introduction

Autism Spectrum Disorder (ASD) is a pervasive developmental disorder arising from neurological developmental abnormalities. Its core characteristics include persistent difficulties in reciprocal social interaction, alongside restricted, repetitive behaviours, interests, or activities. These symptoms emerge during childhood and limit or impair daily functioning (American Psychiatric Association, 2022). Studies on relatives of individuals with ASD find that although they are not diagnosed with ASD, they often exhibit behavioral characteristics similar to those with ASD, albeit not meeting the clinical diagnostic criteria (Xiao et al., 2014; Bailey et al., 1998). Using the Autism-Spectrum Quotient (AQ) in typically developing college students, Baron-Cohen et al. (2001) found that the general population also exhibits cognitive and behavioral features associated with ASD, differing only in degree. Researchers use the concept of autistic traits to describe such characteristics in the population. Autistic traits are a collection of behavioral, personality, and cognitive features associated with ASD (Sucksmith et al., 2011).

Studies reveal that individuals with higher autistic traits experience greater interpersonal difficulties (O’Loghlen & Lang, 2024), including deficits in social attention (Yang et al., 2024), reduced initiative in social interactions, and a lack of knowledge, experience, and behaviors related to social skills (Jin et al., 2020). Additionally, repetitive and stereotyped behaviors are directly linked physiologically to response inhibition abilities (Langen et al., 2012). Using a stop-signal task, Yu (2020) demonstrated that higher autistic traits are associated with poorer response inhibition.

Depression is one of the most common comorbid mental disorders in individuals with autism (Hollocks et al., 2018), and similar associations have been reported for autistic traits (Rai et al., 2018). A self-report study with adult participants found that autistic traits are correlated with depression, self-harm, and suicidal tendencies: higher autistic traits are associated with more severe depression, self-harm, and suicidal tendencies (Sampson et al., 2020). Recent research confirms that impaired interpersonal competence is a key factor in depression among people with autism (Smith & White, 2020; Hedley et al., 2018). Given that individuals with high autistic traits experience interpersonal difficulties, further exploration is needed to determine whether such difficulties lead to increased depression levels.

Previous research has demonstrated that individuals with autism experience heightened levels of depressive mood and symptoms (Hollocks et al., 2018), with impaired interpersonal functioning serving as a significant source of these emotional disturbances (Smith & White, 2020; Hedley et al., 2018). This impairment also makes them more likely to avoid social situations and experience negative emotions and experiences during social interactions (Spence & Rapee, 2016; Dai et al., 2024). The higher an individual’s autistic traits, the more cognitive and behavioral characteristics they exhibit that resemble those of individuals with ASD (Baron-Cohen et al., 2001). Research has also confirmed that increased autistic traits positively influence an individual’s depressive mood and symptoms (Rai et al., 2018; Sampson et al., 2020). Can the depressive mood and symptoms experienced by highly autistic individuals also be attributed to their poorer interpersonal skills, greater social avoidance, and distress? This study will address this core research question through in-depth exploration.

### 1.1. The Relationship Between Autistic Traits and Depression

Meta-analyses find that depression is one of the most common comorbidities in adults with ASD, with current and lifetime prevalence rates of 23–37% (Hollocks et al., 2018). Autistic symptoms negatively impact educational outcomes, employment, and satisfaction with social relationships, and depression exacerbates these impacts (Thiel et al., 2024). Interpersonal difficulties, a core impairment in autism, play a key role in the etiology of depression in adults with autism (Smith & White, 2020; Hedley et al., 2018). More severe autistic symptoms and poorer quality of interpersonal interactions are associated with greater depression (Hedley et al., 2018). A longitudinal study by Rai et al. (2018) on children with ASD and autistic traits found that these children had significantly higher depression symptom scores at age 10 compared to typically developing peers, and this difference persisted until age 18. The researchers identified social communication deficits as an important autism trait contributing to elevated depression symptoms in these children.

Neuroimaging studies indicate structural and functional abnormalities in the amygdala in individuals with ASD, a region regarded as a core node in the “social brain” (Xu et al., 2022). Additionally, research has identified the anterior cingulate cortex (ACC) as a key structure influencing the relationship between ASD and depression. Larger ACC volume is associated with more severe clinician-rated and self-reported autistic symptoms, higher likelihood of self-reported lifetime depression diagnosis, more severe current self-reported depression symptoms, and greater subjective perception of social difficulties (Hao et al., 2025). These findings indicate that interpersonal deficits in ASD have a biological basis and share physiological links with depression.

Individuals with high autistic traits not only experience interpersonal difficulties similar to those with ASD but also exhibit comparable findings regarding depression. In college students (Reed et al., 2016) and general adult samples (Galvin & Richards, 2023), autistic traits correlate with elevated levels of depressive symptoms. Autistic traits also show a significant association with clinical depression (Rai et al., 2018), meaning individuals with high autistic traits tend to have higher levels of clinical depression.

### 1.2. The Relationship Between Interpersonal Competence, Autistic Traits, and Depression

Interpersonal competence refers to an individual’s effectiveness in engaging in interpersonal interactions (O’Loghlen & Lang, 2024). Buhrmester et al. (1988) categorized interpersonal competence into five dimensions: (a) initiating relationships, (b) disclosing personal information, (c) asserting displeasure with others, (d) providing emotional support and advice, and (e) managing interpersonal conflict. Maintaining positive communication and relationships with others is crucial for successful social integration (Yu, 2020). In other words, interpersonal competence serves as a bridge connecting individuals to society as a whole. Individuals with strong interpersonal competence tend to be happier and more energetic (Buhrmester et al., 1988), better able to find meaning in life (R. W. Zhang et al., 2020), and experience less depression, anxiety, and loneliness (Buhrmester et al., 1988; Rodriguez et al., 2015; R. W. Zhang et al., 2020).

Impaired interpersonal competence is a core symptom of ASD. This impairment stems from significant deficits in social cognitive functioning at both behavioral and neural levels (Sasson et al., 2013). Interpersonal difficulties in individuals with high autistic traits are related to social cognitive deficits, and declines in social cognitive abilities and social skills are highly interrelated rather than independent (Sasson et al., 2013). Recent work by Standiford and Hsu (2025) confirmed that individuals with higher autistic traits performed significantly worse on facial expression recognition tasks, indicating deficits in the perception, processing, and interpretation of social information (i.e., facial expressions).

Beyond cognitive deficits, individuals with ASD also exhibit motivational impairments. The social motivation hypothesis suggests that social motivation is a powerful driver of human behavior, and disruptions in social motivational mechanisms may constitute a primary ASD deficit. Specifically, motivational deficits affect the development of social cognition, with social cognitive impairments viewed as a consequence rather than a cause of reduced social interest (Chevallier et al., 2012). Reduced social motivation arises because individuals with ASD derive less reward from social stimuli compared to neurotypical individuals. From early childhood, abnormal reward processing in ASD children leads them to pay less attention to social information (e.g., faces and eye gaze). This reduces opportunities for social learning and hindering the development of social skills (Clements et al., 2018). Spontaneous facial mimicry serves as a physiological indicator of emotional empathy (Meltzoff & Moore, 2002), prompting researchers to investigate the relationship between autistic traits and spontaneous facial mimicry. Sims et al. (2014) conditioned neutral faces with high and low rewards. Then they measured functional connectivity between the ventral striatum (VS; involved in mimicry) and inferior frontal gyrus (IFG; involved in reward processing) while participants viewed happy expressions of these faces. They found significantly stronger VS-IFG connectivity during high-reward happy faces compared to low-reward conditions, but this difference was negatively correlated with autistic traits. In other words, individuals with high autistic traits may have impaired connectivity between mimicry and reward systems, leading to deficits in facial mimicry abilities.

Individuals with high autistic traits exhibit widespread and persistent interpersonal impairments similar to those with ASD, particularly difficulties in processing and responding to complex interpersonal cues in reciprocal emotional interactions (e.g., knowing when and how to join a conversation, or what topics to avoid; American Psychiatric Association, 2022). This deficit results in poor interpersonal competence and varying degrees of social distress (O’Loghlen & Lang, 2024). As noted earlier, individuals with poor interpersonal competence experience more negative emotions, including depression.

Furthermore, Dai et al. (2024) found through experimental research involving participants from China and the United States that social anxiety impairs individuals’ interpersonal competence. And interpersonal competence predicts outcomes of social anxiety treatment (Spence & Rapee, 2016).

### 1.3. The Relationship Between Social Avoidance and Distress, Autistic Traits, and Depression

Social avoidance and distress refer to an individual’s tendency to avoid social interactions and the experience of negative emotions such as distress during social engagement (Watson & Friend, 1969). Avoidance is a behavioral manifestation and distress is an emotional experience (Du, 2023). Social avoidance and distress are key components of social anxiety (Watson & Friend, 1969; X. Li et al., 2023). Social anxiety disorder is characterized by avoidance of social situations, fear of negative evaluation, and poor interpersonal skills. This is consistent with the clinical manifestations of ASD (American Psychiatric Association, 2022) and the symptoms of social avoidance and distress. X. Li et al. (2023) found that non-autistic individuals with high autistic traits are more likely to exhibit social avoidance and distress; Ujiie and Takahashi (2022) showed that greater autistic traits correlate with higher aversion to social contact. In short, more pronounced autistic traits are associated with greater social avoidance and distress.

Research indicates that social anxiety disorder frequently co-occurs with major depressive disorder (American Psychiatric Association, 2022). As subcomponents of social anxiety, social avoidance and distress show a significant positive correlation with depression levels in college students (Tu et al., 2025; Yuan et al., 2022) and directly influence these levels (Yuan et al., 2022). Individuals with severe social avoidance encounter numerous problems in peer interactions (Ding et al., 2018). Frequent avoidance behaviors prevent them from adapting to group life or lead to social neglect, resulting in intense negative emotional experiences (Hill et al., 2019).

In summary, individuals with high autistic traits have poor interpersonal competence (Standiford & Hsu, 2025), which increases their risk of greater social avoidance and distress (Dai et al., 2024). Meanwhile, social distress and avoidance elevate depression levels in college students (Tu et al., 2025; Yuan et al., 2022). However, no studies have comprehensively examined the roles of interpersonal competence and social avoidance and distress in the relationship between autistic traits and depression. Therefore, this study hypothesizes that interpersonal competence, and social avoidance and distress play a chained mediating role in the relationship between autistic traits and depression.

## 2. Materials and Methods

### 2.1. Participants and Ethics

Data collection for this study was conducted online. Before formally answering, participants were required to read the informed consent form. Data collection proceeded only after participants demonstrated understanding and agreement with the consent form’s contents. If a participant declined to consent, data collection was terminated. 

We gathered primary data through Wenjuanxing (http://www.wjx.cn, accessed on 31 July 2024), receiving a total of 804 completed questionnaires. Respondents who completed the questionnaire in less than 360 s or answered the lie detection questions incorrectly were deemed not to have responded seriously, and their data were excluded. After excluding invalid questionnaires, 598 valid responses remained, yielding a response rate of 73%. The sample comprised 17.4% males and 82.6% females (*M*_age_ = 20.14 years, *SD*_age_ = 4.39). Given the low male representation in the initial data, a second data collection was conducted online (TCLab, https://www.testcloudlab.com/testcloud-study/index, accessed on 6 November 2025), yielding data from 76 male participants. Thus, the study yielded a total of 674 valid responses, with males comprising 26.7% and females 73.3% (*M*_age_ = 20.22 years, *SD*_age_ = 2.11).

Ethical approval for this study was confirmed through the [I can’t read you: Inefficient perception of social cues in individuals with high autism traits application] review process by the University Ethics Committee in [April, 2024]. Formal IRB certification (protocol code: 2025090802) was obtained subsequent to data collection to fulfill specific journal requirements. All participants signed informed consent. In this study, we did not use generative artificial intelligence (GenAI) to generate text, data, or graphics, or to assist in the study design, data collection, analysis, or interpretation.

### 2.2. Research Instruments

#### 2.2.1. Autistic Traits

This study used the Chinese version of the Autism-Spectrum Quotient (AQ), revised by L. Zhang and Wang (2014) from the original scale developed by Baron-Cohen et al. (2001). The questionnaire contains 50 items. Each item has 4 response options: “definitely disagree,” “slightly disagree,” “slightly agree,” and “definitely agree.” A binary scoring system was used in the original questionnaire: “definitely agree” or “slightly agree” scored 1 point, while “strongly disagree” or “slightly disagree” scored 0 points. Twenty-five items were reverse-scored. Higher scores indicate higher autistic traits. In this study, the Cronbach’s α calculated using a 0–1 scoring system was 0.62, which is comparable to Cronbach’s α values reported in similar studies employing this questionnaire. For instance, Lin (2014) reported Cronbach’s α = 0.62, H. Wu and Liu (2024) reported Cronbach’s α = 0.64, while H. Liu and Wang (2020) found Cronbach’s α = 0.63. However, when we calculated the Cronbach’s α using a 4-point Likert scale, the result was 0.71. This suggests that employing a 4-point scoring method may be more appropriate when using this questionnaire.

#### 2.2.2. Interpersonal Competence

The Interpersonal Competence Questionnaire (ICQ) was developed by psychologists at the University of California, Los Angeles (Buhrmester et al., 1988). The questionnaire includes 40 items, using a 5-point Likert scale. The Chinese version has good reliability (Cronbach’s α = 0.87) and construct validity (Y. Wei, 2005). In this study, Cronbach’s α = 0.93.

#### 2.2.3. Social Avoidance and Distress

The Social Avoidance and Distress Scale (SAD), developed by Watson and Friend (1969) and revised by Wang et al. (1999), consists of 28 items, using a yes/no scoring format. The scale’s Cronbach’s α = 0.85. In this study, Cronbach’s α = 0.92.

#### 2.2.4. Depression

The 13-item Beck Depression Inventory (BDI), developed by Beck in 1974, uses a 0–3 scoring system. Total scores distinguish the presence and severity of depressive symptoms (W. Y. Wu, 2005). The scale has a Cronbach’s α coefficient of 0.718 (Tan et al., 2011). In this study, Cronbach’s α = 0.88.

### 2.3. Data Analysis

Using SPSS 27.0, we performed data preprocessing, descriptive statistics, and correlation analysis on the four variables: the independent variable (autistic traits), the dependent variable (depression), and the mediating variables (interpersonal skills, social avoidance, and distress). The Process 4.1 plugin was used for mediation analysis and moderation analysis. 

## 3. Results

### 3.1. Common Method Bias Test

Harman’s single-factor test was used to examine potential common method bias. Results showed 32 factors with eigenvalues greater than 1, and the first factor explained 16.22% of the total variance, which is less than 40%. Thus, no serious common method bias was found in this study.

### 3.2. Descriptive Statistics and Correlation Analysis

Table 1 presents the means, standard deviations, and correlation analysis results for all variables. Autistic traits, interpersonal competence, social avoidance and distress, and depression showed significant overall correlations: autistic traits were positively correlated with social avoidance and distress and depression; social avoidance and distress was positively correlated with depression; all other variable pairs showed negative correlations.

These results align with previous research, indicating that higher autistic traits are associated with poorer interpersonal competence, greater social avoidance and distress, and more severe depressive symptoms. Poorer interpersonal competence correlates with greater social avoidance and distress and more severe depression, while greater social avoidance and distress is associated with more severe depression.

### 3.3. Mediation Effect Test

**Figure 1:**
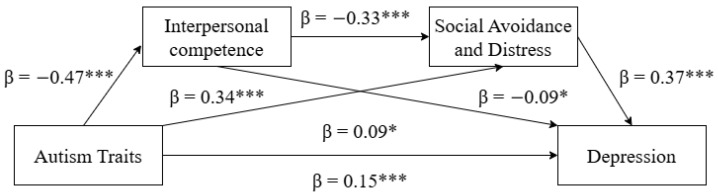
Interpersonal competence, and social avoidance and distress as the mediating variables between autistic traits and depression. Standardized coefficients are presented. Note: * *p* < 0.05, *** *p* < 0.001.

This study used Model 6 in the Process 4.1 plugin for SPSS 27.0 (Hayes, 2018), with 5000 bootstrap samples and a 95% confidence interval. Controlling for gender and age, a chained mediation analysis was conducted with autistic traits as the independent variable, interpersonal competence and social avoidance and distress as mediating variables, and depression as the dependent variable. The overall regression equation was significant (*R*^2^ = 0.10, *F* = 72.55, *p* < 0.001). As shown in Figure 1, autistic traits had a significant negative effect on interpersonal competence (β = −0.47, *t* = −13.81, *p* < 0.001); interpersonal competence had a significant negative effect on social avoidance and distress (β = −0.33, *t* = −9.25, *p* < 0.001); social avoidance and distress had a significant positive effect on depression (β = 0.37, *t* = 8.85, *p* < 0.001). Additionally, autistic traits had a direct effect on depression (β = 0.09, *t* = 2.10, *p* < 0.05). Mediation effect test results are presented in Figure 1 and Table 2 and Table 3. Specifically, the chained mediation effect of interpersonal competence and social avoidance/distress consisted of three indirect paths: (1) autistic traits → interpersonal competence → depression (0.02); (2) autistic traits → social avoidance and distress → depression (0.06); (3) autistic traits → interpersonal competence → social avoidance and distress → depression (0.03). These indirect effects accounted for 13.33%, 40.00%, and 20.00% of the total effect, respectively.

### 3.4. Testing the Moderated Mediating Effect

Given the significant gender ratio disparity in this study, gender was incorporated as a moderator variable to examine whether it influences the model outcomes.

Gender was added as a moderator to the chained mediation model equation, and Process Model 83 was run. Results are presented in Table 4. Equation (1) was not significant; the interaction term between autistic traits and gender did not significantly predict interpersonal skills (β = 0.06, SE = 0.15, *p* > 0.05). That is, gender did not moderate the initial chain mediation path (autistic traits → interpersonal skills).

## 4. Discussion

This study used a mediation model to examine the relationship between autistic traits and depression, as well as the role of interpersonal competence, and social avoidance and distress in this relationship. It was found that autistic traits are positively related to depression, with interpersonal competence and social avoidance and distress playing mediating roles. These results provide valuable insights for identifying ways to reduce depression levels in individuals with autistic traits. 

### 4.1. The Relationship Between Individual Autistic Traits and Depression Levels

This study found a positive relationship between individual autistic traits and depression, which is consistent with previous research (Reed et al., 2016; Galvin & Richards, 2023; Rai et al., 2018). Sampson et al. (2020) demonstrated that autistic traits are associated with adverse clinical outcomes, including increased depressive symptoms, self-harm behaviors, and suicidal tendencies. The results of this study revealed that autistic traits can directly predict depression levels and also influence depression through indirect pathways, with interpersonal competence and social avoidance and social distress serving as important intermediate variables in this process. This is consistent with previous findings that individuals with ASD can affect depression levels through both direct and indirect pathways (van Heijst et al., 2020).

### 4.2. Correlations Among Variables

This study found that autistic traits were negatively correlated with interpersonal skills and positively correlated with social avoidance and distress. These findings align with prior research, indicating that individuals with higher autistic traits exhibit poorer interpersonal skills (C. Liu et al., 2024) and are more prone to social avoidance and distress, and depressive symptoms (Dai et al., 2024; Ujiie & Takahashi, 2022). Furthermore, higher autistic traits correlate with lower levels of theory of mind (Baron-Cohen et al., 2001), potentially impairing individuals’ ability to recognize others’ emotions and intentions. This, in turn, hinders effective social responses and manifests as diminished social competence. Naturally, such social difficulties may lead to social avoidance and distress, manifesting as a significant positive correlation between autistic traits and social avoidance and distress. Individuals with strong interpersonal skills receive ample support and positive feedback in social settings, experiencing less depression (Buhrmester et al., 1988; Rodriguez et al., 2015; R. W. Zhang et al., 2020). Individuals with high levels of social avoidance and distress lose a major source of social support while being unable to completely avoid social situations, leading to increased depressive symptoms (Tu et al., 2025; Yuan et al., 2022).

### 4.3. The Mediating Role of Interpersonal Competence, and Social Avoidance and Distress Between Individual Autistic Traits and Depression Levels

The results of this study showed that interpersonal competence, and social avoidance and distress exert separate mediating effects between autistic traits and depression. Interpersonal competence mediates the relationship between autistic traits and depression. Individuals with high autistic traits often exhibit difficulties in social emotions and communication, including deficits in emotion regulation and interpersonal competence (Vaiouli & Panayiotou, 2021). The Empathizing–Systemizing (E-S) theory suggests that the social and communication impairments in ASD arise from deficits in empathy, while non-social characteristics such as narrow interests, stereotyped behaviors, and “islands of ability” result from intact or even superior systematizing abilities (Baron-Cohen, 2009; Qi & Chen, 2013). Baron-Cohen (2009) proposed that empathy consists of cognitive and responsive components: the cognitive component involves recognizing one’s own and others’ emotions and feelings, while the responsive component requires appropriate reactions to these emotions. Similar to individuals with ASD, those with high autistic traits have impairments in both components of empathy. This may stem from the abnormal sensory experiences and interests toward environmental stimuli observed in 82–97% of individuals with autism (Dellapiazza et al., 2018). The intense theory of mind in autism posits that the core manifestation of autism spectrum disorders is an overly intense perception, attention, memory, and emotional response to sensory information (Markram et al., 2007). Research indicates that individuals with heightened sensitivity exhibit distinct patterns of gaze toward emotional faces compared to neurotypical individuals, thereby influencing recognition outcomes (Christou et al., 2024). This, in turn, negatively impacts the interpersonal competence of individuals with autistic traits, making it difficult for them to establish healthy relationships, which in turn leads to depressive emotions (Buhrmester et al., 1988; Rodriguez et al., 2015; R. W. Zhang et al., 2020). Existing research has identified a positive correlation between sensitivity to environmental stimuli and autistic traits (Assary et al., 2024), providing some support for this hypothesis.

Social avoidance and distress mediate the relationship between autistic traits and depression. Anxiety is considered a bridge between autism and depression, with social anxiety being particularly relevant to depressive emotions (van Heijst et al., 2020). Individuals with high social anxiety are more likely to use inappropriate coping strategies in social activities, leading to negative social outcomes (B. Li et al., 2003) and subsequent depressive emotions. Liao (2023) summarized three types of internal factors influencing social avoidance and distress: cognitive dissonance, low self-evaluation, and personality traits. Individuals with high autistic traits tend to underestimate their ability to judge the intensity of emotional experiences and lack confidence in their social skills—that is, they hold a low self-evaluation of their own interpersonal competence. As a personality trait, autistic traits are characterized by core deficits in social interaction, which prominently manifest as reduced initiative in social interaction, avoidance of social interaction, and distress from social interaction pressure (American Psychiatric Association, 2022). Therefore, individuals with high autistic traits exhibit more frequent social avoidance and distress, which further increases their depression levels (Tu et al., 2025; Yuan et al., 2022).

### 4.4. The Chained Mediation of Interpersonal Competence, and Social Avoidance and Distress

This study found that interpersonal competence, and social avoidance and distress play a chained mediating role between autistic traits and depression. Individuals with high autistic traits are generally considered to have difficulties in theory of mind (Brewer et al., 2023), which, in the E-S theory, is equivalent to the cognitive component of empathy (Baron-Cohen, 2009). As an indispensable factor in interpersonal interactions (Miller & Eisenberg, 1988), empathy positively influences interpersonal competence (Tian, 2015). Individuals with high empathy can keenly perceive others’ emotional needs through subtle cues and maintain harmonious relationships (Johnson et al., 2009). However, individuals with high autistic traits often have significant deficits in interpersonal competence, rendering them less able to navigate complex and dynamic social contexts and to establish and sustain stable relationships, thereby leading to social avoidance and distress. Moreover, Christou et al. (2024) found that individuals across different age groups exhibit distinct patterns of visual attention allocation toward various emotional facial expressions. This phenomenon may manifest differently in individuals with autism and those with high autistic traits compared to typically developing individuals. A study on treating social anxiety disorder with different methods found that greater use of interpersonal therapy at the beginning of treatment leads to better outcomes, and training in interpersonal competence is a central component of such therapy (Sinai et al., 2012). Epkins and Heckler (2011) indicated that improving adolescents’ interpersonal relationships, including enhancing interpersonal competence, is a major aspect of treating social anxiety disorder. Tu et al. (2025) found that interpersonal competence training can effectively reduce individuals’ social avoidance behaviors. As mentioned earlier, social distress and avoidance are key components of social anxiety, and deficits in interpersonal competence also contribute to social avoidance and distress. Consistent with previous studies (Tu et al., 2025; Yuan et al., 2022), social avoidance and distress in college students are significantly positively correlated with depression and positively predict depression levels. Social avoidance and distress consist of two components: social avoidance is a behavioral manifestation (Du, 2023) that isolates individuals from normal social interactions, depriving them of social support and recognition; distress is an emotional experience (Du, 2023) that accumulates negative emotions. Over time, the lack of outlets for negative emotions and the increased accumulation lead to higher levels of depression compared to the general population. Therefore, more attention should be paid to the mental health of individuals with high autistic traits, especially since they tend to underestimate their ability to judge emotional experiences and struggle with emotional expression (Huggins et al., 2020), which may have more severe negative impacts on their mental health. 

### 4.5. Research Limitations and Future Directions

In this study, the sample exhibited significant gender differences. Although we examined whether gender moderated the findings and found no moderating effect, this does not entirely rule out the influence of gender differences. For instance, prior research indicates that female autistic individuals exhibit greater social camouflage and demonstrate superior social skills compared to males (Gould & Ashton-Smith, 2011). Thus, gender differences may have influenced the findings of this study. Additionally, while Cronbach’s α was relatively low in our findings, substantial variations emerged when using different scoring methods. This suggests that future research should explore more appropriate scoring approaches for the AQ tool when applied to Chinese university students.

The Individual-Environment Congruence Hypothesis (Sagiv & Schwartz, 2000) posits that if a cultural environment provides diverse opportunities to fulfill goals embodied in cultural norms, while simultaneously reinforcing the importance of such norms, then individuals whose values align with these societal norms should exhibit a positive correlation with adaptive outcomes—regardless of the specific content of those norms or goals. Chinese culture suppresses the free expression of emotions, viewing emotional display as detrimental to interpersonal harmony (M. F. Wei et al., 2013). Consequently, individuals living within a Chinese cultural context tend to express emotions more inwardly (Chang & Fang, 2018). This necessitates individuals relying on more subtle social cues to discern the state (e.g., emotions) of their social partners. However, individuals with high autistic traits exhibit abnormal attention to social cues (Yang et al., 2024), which may hinder their perception of such information and consequently lead to interpersonal difficulties.

In summary, autistic traits are closely related to depression, with interpersonal competence and social avoidance and distress playing mediating roles. These findings suggest that improving individuals’ social abilities can enhance their mental health when exploring the connection between autistic traits and depression. Yin et al. (2022) conducted theory of mind training for individuals with high autistic traits. Employing a group counseling approach, the intervention first involved cognitive training in emotion recognition and expression, followed by emotional management and perspective-taking interventions. Finally, it included training in listening and confiding skills, along with behavioral interventions focused on empathy and viewpoint adoption. The group counseling program spanned eight weeks. Results showed that the intervention significantly improved their perspective-taking abilities while markedly reducing their social avoidance and distress.

## 5. Conclusions

The findings of this study reveal a significant positive correlation between autistic traits and depression in individuals. Autistic traits influence depressive levels through interpersonal competence and social avoidance and distress. This suggests that future research should explore the underlying mechanisms linking autistic traits to social functioning while also focusing on improving mental health by enhancing individuals’ interpersonal competence.

## Figures and Tables

**Table 1:** Descriptive statistics and correlations between variables.

	1	2	3	4
1. Autism-Spectrum quotient	-			
2. Interpersonal competence questionnaire	−0.47 ***	-		
3. Social avoidance and distress scale	0.50 ***	−0.49 ***	-	
4. Beck depression inventory	0.31 ***	−0.31 ***	0.46 ***	-
*M*	119.23	135.78	15.01	5.27
*SD*	10.30	19.45	7.75	5.20

*** *p* < 0.001.

**Table 2:** Results of the Mediated Effect Test.

	Interpersonal Competence		Social Avoidance and Distress		Depression	
	β	*t*	β	*t*	β	*t*
AT	−0.47	−13.81 ***	0.26	9.54 ***	0.09	2.10 *
Interpersonal Competence			−0.33	−9.25 ***	−0.09	−2.22 *
Social Avoidance and Distress					0.37	8.85 ***
*R* ^2^	0.22		0.33		0.22	
*F*	190.75 ***		166.47 ***		64.28 ***	

AT: autistic traits. * *p* < 0.05; *** *p* < 0.001.

**Table 3:** The chained mediating role of interpersonal competence, social avoidance and distress in the influence of autistic traits on depression.

Indirect Effects	Effect	Proportion of the Total Effect	BootSE	95% CI
AT → Depression	0.04	26.67%		[0.00, 0.08]
AT → Interpersonal Competence → Depression	0.02	13.33%	0.01	[0.00, 0.04]
AT → Social Avoidance and Distress → Depression	0.6	40.00%	0.01	[0.05, 0.09]
AT → Interpersonal Competence → Social Avoidance and Distress → Depression	0.03	20.00%	0.01	[0.02, 0.04]
AT → Depression (Total indirect effect)	0.11	73.33%	0.02	[0.09, 0.15]

AT: autistic traits.

**Table 4:** Testing for Moderated Mediating Effects.

	Equation (1) (Dependent Variable: Interpersonal Competence)		Equation (2) (Dependent Variable: Social Avoidance and Distress)		Equation (3) (Dependent Variable: Depression)	
	β	*t*	β	*t*	β	*t*
Gender	2.09	1.28				
Autistic Traits	−0.91	−12.09 ***	0.26	9.54 ***	0.04	2.10 *
Autistic Traits × Gender	0.05	−0.36				
Interpersonal Competence			−0.13	−9.25 ***	−0.02	−2.22 *
Social Avoidance and Distress					0.25	8.85 ***
*R* ^2^	0.22		0.33		0.22	
*F*	64.31 ***		166.47 ***		64.28 ***	

* *p* < 0.05; *** *p* < 0.001.

## Data Availability

The data that support the findings of this study are available from the corresponding author upon reasonable request.

## Abbreviations

The following abbreviations are used in this manuscript:

ACC	Anterior cingulate cortex
AQ	Autism-Spectrum quotient
ASD	Autism spectrum disorder
AT	Autistic traits
BDI	Beck depression inventory
ICQ	Interpersonal competence questionnaire
IFG	Inferior frontal gyrus
SAD	Social avoidance and distress scale
VS	Ventral striatum

## References

[B1-behavsci-15-01658] American Psychiatric Association (2022). Diagnostic and statistical manual of mental disorders fifth edition DSM-5-TR.

[B2-behavsci-15-01658] Assary E., Oginni O. A., Morneau-Vaillancourt G., Krebs G., Peel A. J., Palaiologou E., Lockhart C., Ronald A., Eley T. C. (2024). Genetics of environmental sensitivity and its association with variations in emotional problems, autistic traits, and wellbeing. Molecular Psychiatry.

[B3-behavsci-15-01658] Bailey A., Palferman S., Heavey L., Le Couteur A. (1998). Autism: The phenotype in relatives. Journal of Autism and Developmental Disorders.

[B4-behavsci-15-01658] Baron-Cohen S. (2009). Autism: The empathizing-systemizing (E-S) theory. Annals of the New York Academy of Sciences.

[B5-behavsci-15-01658] Baron-Cohen S., Wheelwright S., Skinner R., Martin J., Clubley E. (2001). The autism-spectrum quotient (AQ): Evidence from Asperger syndrome/high-functioning autism, males and females, scientists and mathematicians. Journal of Autism and Developmental Disorders.

[B6-behavsci-15-01658] Brewer N., Lucas C. A., Lim A., Young R. L. (2023). Detecting dodgy behaviour: The role of autism, autistic traits and theory of mind. Autism.

[B7-behavsci-15-01658] Buhrmester D., Furman W., Wittenberg M. T., Reis H. T. (1988). Five domains of interpersonal competence in peer relationships. Journal of Personality & Social Psychology.

[B8-behavsci-15-01658] Chang B., Fang J. (2018). The association between ambivalence over emotion expression and psychological symptoms in cross-cultural studies: A moderated mediation model. Journal of Psychological Science.

[B9-behavsci-15-01658] Chevallier G., Troiani K. V., Brodkin E. S., Schultz R. T. (2012). The social motivation theory of autism. Trends in Cognitive Sciences.

[B10-behavsci-15-01658] Christou A. I., Fanti K., Mavrommatis I., Soursou G., Eliadi E. (2024). Parental sensory processing sensitivity predicts children’s visual scanning pattern of emotional faces. Journal of Psychopathology and Behavioral Assessment.

[B11-behavsci-15-01658] Clements C. C., Zoltowski A. R., Yankowitz L. D., Yerys B. E., Schultz R. T., Herrington J. D. (2018). Evaluation of the social motivation hypothesis of autism: A systematic review and meta-analysis. JAMA Psychiatry.

[B12-behavsci-15-01658] Dai Y., Jiang T., Wildschut T., Sedikides C. (2024). Nostalgia counteracts social anxiety and enhances interpersonal competence. Social Psychological and Personality Science.

[B13-behavsci-15-01658] Dellapiazza F., Vernhet C., Blanc N., Miot S., Schmidt R., Baghdadli A. (2018). Links between sensory processing, adaptive behaviours, and attention in children with autism spectrum disorder: A systematic review. Psychiatry Research.

[B14-behavsci-15-01658] Ding X. C., Deng X. M., Sang B., Li D. (2018). Relations between social avoidance and peer problems in Chinese early adolescents: A moderated-mediation model. Psychological Development and Education.

[B15-behavsci-15-01658] Du J. M. (2023). Effects of high-definition transcranial direct current stimulation on social avoidance in high autism traits individuals. Master’s thesis.

[B16-behavsci-15-01658] Epkins C. C., Heckler D. R. (2011). Integrating etiological models of social anxiety and depression in youth: Evidence for a cumulative interpersonal risk model. Clinical Child And Family Psychology Review.

[B17-behavsci-15-01658] Galvin J., Richards G. (2023). The indirect effect of self-compassion in the association between autistic traits and anxiety/depression: A cross-sectional study in autistic and non-autistic adults. Autism.

[B18-behavsci-15-01658] Gould J., Ashton-Smith J. (2011). Missed diagnosis or misdiagnosis? Girls and women on the autism spectrum. Good Autism Practice (GAP).

[B19-behavsci-15-01658] Hao Y., Banker S., Trayvick J., Barkley S., Peters A. W., Thinakaran A., McLaughlin C., Gu X., Schiller D., Foss-Feig J. (2025). Understanding depression in autism: The role of subjective perception and anterior cingulate cortex volume. Molecular Autism.

[B20-behavsci-15-01658] Hayes A. F. (2018). Introduction to mediation, moderation, and conditional process analysis: A regression-based approach.

[B21-behavsci-15-01658] Hedley D., Uljarević M., Foley K.-R., Richdale A., Trollor J. (2018). Risk and protective factors underlying depression and suicidal ideation in autism spectrum disorder. Depression and Anxiety.

[B22-behavsci-15-01658] Hill M. S., Yorgason J. B., Elson L. J., Jensen A. C. (2019). Social withdrawal and loneliness among older adult athletes: A case for playing alone. Journal of Aging and Physical Activity.

[B23-behavsci-15-01658] Hollocks M. J., Lerh J. W., Magiati I., Meiser-Stedman R., Brugha T. S. (2018). Anxiety and depression in adults with autism spectrum disorder: A systematic review and meta-analysis. Psychological Medicine.

[B24-behavsci-15-01658] Huggins C. F., Cameron I. M., Williams J. H. G. (2020). Autistic traits predict underestimation of emotional abilities. Journal of Experimental Psychology: General.

[B25-behavsci-15-01658] Jin Y., Chen X., Zhao X. (2020). Autistic traits and social skills in Chinese college students: Mediating roles of adult attachment styles and empathy. Current Psychology.

[B26-behavsci-15-01658] Johnson S. A., Filliter J. H., Murphy R. R. (2009). Discrepancies between self- and parent-perceptions of autistic traits and empathy in high functioning children and adolescents on the autism spectrum. Journal of Autism and Developmental Disorders.

[B27-behavsci-15-01658] Langen M., Leemans A., Johnston P., Ecker C., Daly E., Murphy C. M., Dell’acqua F., Durston S., AIMS Consortium, Murphy D. G. (2012). Fronto-striatal circuitry and inhibitory control in autism: Findings from diffusion tensor imaging tractography. Cortex.

[B28-behavsci-15-01658] Li B., Zhong J., Qian M. Y. (2003). Regression analysis on social anxiety proneness among college students. Chinese Mental Health Journal.

[B29-behavsci-15-01658] Li X., Shen H., Kong H., Xie J. (2023). Autistic traits predict social avoidance and distress: The chain mediating role of perceived stress and interpersonal alienation. Scandinavian Journal of Psychology.

[B30-behavsci-15-01658] Liao J. (2023). Relationship between relative deprivation and social avoidance or distress of rural left-behind junior middle school students: A moderated mediation model. Master’s thesis.

[B31-behavsci-15-01658] Lin G. (2014). Pilot study of the autism spectrum quotient among Chinese college students. Journal of Changchun University of Technology (Higher Education Study Edition).

[B32-behavsci-15-01658] Liu C., Zhang Q., Liu Y., Wang Z., Chen F., Li Y., Zhao Y., Zhu J., Li D., Zhu C. (2024). The association between autistic traits and depression in college students: The mediating roles of interpersonal emotion regulation and social self-efficacy. Psychology Research and Behavior Management.

[B33-behavsci-15-01658] Liu H., Wang W. (2020). Autistic traits and suicidal ideation: A moderate mediation model. Chinese Journal of Clinical Psychology.

[B34-behavsci-15-01658] Markram H., Rinaldi T., Markram K. (2007). The intense world syndrome—An alternative hypothesis for autism. Frontiers in Neuroscience.

[B35-behavsci-15-01658] Meltzoff A. N., Moore M. K. (2002). Imitation, memory, and the representation of persons. Infant Behavior and Development.

[B36-behavsci-15-01658] Miller P. A., Eisenberg N. (1988). The relation of empathy to aggressive and externalizing/antisocial behavior. Psychological Bulletin.

[B37-behavsci-15-01658] O’Loghlen J. J., Lang C. P. (2024). High autistic traits or low social competence? Correlates of social camouflaging in non-autistic adults. Autism in Adulthood.

[B38-behavsci-15-01658] Qi X. L., Chen W. (2013). The pendulum tilted to the “Truth”: Empathizing-Systemizing theory in autism. Journal of Psychological Science.

[B39-behavsci-15-01658] Rai D., Culpin I., Heuvelman H., Magnusson C. M., Carpenter P., Jones H. J., Emond A. M., Zammit S., Golding J., Pearson R. M. (2018). Association of autistic traits with depression from childhood to age 18 years. JAMA Psychiatry.

[B40-behavsci-15-01658] Reed P., Giles A., Gavin M., Carter N., Osborne L. A. (2016). Loneliness and social anxiety mediate the relationship between autism quotient and quality of life in university students. Journal of Developmental and Physical Disabilities.

[B41-behavsci-15-01658] Rodriguez E. M., Donenberg G. R., Emerson E., Wilson H. W., Javdani S. (2015). Externalizing symptoms moderate associations among interpersonal skills, parenting, and depressive symptoms in adolescents seeking mental health treatment. Journal of Youth and Adolescence.

[B42-behavsci-15-01658] Sagiv L., Schwartz S. H. (2000). Value priorities and subjective well-being: Direct relations and congruity effects. European Journal of Social Psychology.

[B43-behavsci-15-01658] Sampson K. N., Upthegrove R., Abu-Akel A., Haque S., Wood S. J., Reniers R. (2020). Co-occurrence of autistic and psychotic traits: Implications for depression, self-harm and suicidality. Psychological Medicine.

[B44-behavsci-15-01658] Sasson N. J., Nowlin R. B., Pinkham A. E. (2013). Social cognition, social skill, and the broad autism phenotype. Autism.

[B45-behavsci-15-01658] Sims T. B., Neufeld J., Johnstone T., Chakrabarti B. (2014). Autistic traits modulate frontostriatal connectivity during processing of rewarding faces. Social Cognitive and Affective Neuroscience.

[B46-behavsci-15-01658] Sinai D., Gur M., Lipsitz J. D. (2012). Therapist adherence to interpersonal vs. supportive therapy for social anxiety disorder. Psychotherapy Research.

[B47-behavsci-15-01658] Smith I. C., White S. W. (2020). Socio-emotional determinants of depressive symptoms in adolescents and adults with autism spectrum disorder: A systematic review. Autism.

[B48-behavsci-15-01658] Spence S. H., Rapee R. M. (2016). The etiology of social anxiety disorder: An evidence-based model. Behaviour Research and Therapy.

[B49-behavsci-15-01658] Standiford B. J., Hsu K. J. (2025). Autistic traits, alexithymia, and emotion recognition of human and anime faces. Development and Psychopathology.

[B50-behavsci-15-01658] Sucksmith E., Roth I., Hoekstra R. A. (2011). Autistic traits below the clinical threshold: Reexamining the broader autism phenotype in the 21st century. Neuropsychology Review.

[B51-behavsci-15-01658] Tan L., Zhang T. T., Wang Z. Y., Xu B. W. (2011). Application of the Beck self-rating depression scale among patients with infertility. Maternal and Child Health Care of China.

[B52-behavsci-15-01658] Thiel T., Riedelbauch S., Gaigg S., Roessner V., Ring M. (2024). The impact of depressive and anxious symptoms on quality of life in adults on the autism spectrum. Autism Research.

[B53-behavsci-15-01658] Tian J. (2015). The relationship between empathy and interpersonal skills of class psychological commissioners and its intervention study. Master’s thesis.

[B54-behavsci-15-01658] Tu Y., Su Y., Yang K., Jin L., Li B., Chen W., Yuan Y., Wu D. (2025). The role of depression and interpersonal trust between anxiety and social avoidance among college students: A structural equation model. Current Psychology.

[B55-behavsci-15-01658] Ujiie Y., Takahashi L. (2022). Associations between self-reported social touch avoidance, hypersensitivity, and autistic traits: Results from questionnaire research among typically developing adults. Personality and Individual Differences.

[B56-behavsci-15-01658] Vaiouli P., Panayiotou G. (2021). Alexithymia and autistic traits: Associations with social and emotional challenges among college students. Frontiers in Neuroscience.

[B57-behavsci-15-01658] van Heijst B. F. C., Deserno M. K., Rhebergen D., Geurts H. M. (2020). Autism and depression are connected: A report of two complimentary network studies. Autism.

[B58-behavsci-15-01658] Wang X. D., Wang X. L., Ma H. (1999). Rating scales for mental health.

[B59-behavsci-15-01658] Watson D., Friend R. (1969). Measurement of social-evaluative anxiety. Journal of Consulting & Clinical Psychology.

[B60-behavsci-15-01658] Wei M. F., Su J. C., Carrera S., Lin S. P., Yi F. (2013). Suppression and interpersonal harmony: A cross-cultural comparison between Chinese and European Americans. Journal of Counseling Psychology.

[B61-behavsci-15-01658] Wei Y. (2005). Reliability and validity of the interpersonal competence questionnaire in college students. Chinese Journal of School Health.

[B62-behavsci-15-01658] Wu H., Liu J. (2024). Varies from person to person: The effect of interpersonal sensitivity on somatization of autistic college students. Psychological Exploration.

[B63-behavsci-15-01658] Wu W. Y. (2005). Handbook of behavior medical scales.

[B64-behavsci-15-01658] Xiao X., Yang N., Qian L. Q., Zhou S. J. (2014). Personality, empathy and broad autism phenotype of parents of the children with autism. Chinese Journal of Clinical Psychology.

[B65-behavsci-15-01658] Xu L., Zheng X., Yao S., Li J., Fu M., Li K., Zhao W., Li H., Becker B., Kendrick K. M. (2022). The mirror neuron system compensates for amygdala dysfunction-associated social deficits in individuals with higher autistic traits. NeuroImage.

[B66-behavsci-15-01658] Yang F., Tian J., Yuan P., Liu C., Zhang X., Yang L., Jiang Y. (2024). Unconscious and conscious gaze-triggered attentional orienting: Distinguishing innate and acquired components of social attention in children and adults with autistic traits and autism spectrum disorders. Research.

[B67-behavsci-15-01658] Yin Z., Zhao H., Xuan B. (2022). The effect of sub-threshold autistic traits on social avoidance and distress in high school freshmen: The role of perspective taking and an intervention study. Chinese Journal of Behavioral Medicine and Brain Science.

[B68-behavsci-15-01658] Yu Y. X. (2020). The brain mechanism of autistic traits in college students and its influence factors. Doctoral dissertation.

[B69-behavsci-15-01658] Yuan Y., Jiang S., Wen X., Han Z., Wu D., Wang X., Ye T., Hu Y., Jeong J., Xiang M. (2022). The chain-mediation pathway of social avoidance to depression in college students is regulated by self-esteem. Frontiers in Psychology.

[B70-behavsci-15-01658] Zhang L., Wang K. (2014). Reliability and validity of the Chinese version of the autism-spectrum quotient [Article]. National Mental Health Academic Conference.

[B71-behavsci-15-01658] Zhang R. W., Ke S. J., Lian R., Li D. (2020). The association between interpersonal competence and meaning in life: Roles of loneliness and grade. Psychological Development and Education.

